# Recent advances of novel targeted therapy in non-small cell lung cancer

**DOI:** 10.1186/1756-8722-2-2

**Published:** 2009-01-21

**Authors:** Jed A Katzel, Michael P Fanucchi, Zujun Li

**Affiliations:** 1 Department of Hematology and Oncology, Saint Vincent's Hospital, Manhattan and New York Medical College, Valhalla, NY, USA

## Abstract

Lung cancer is the leading cause of cancer deaths world-wide. Recent advances in cancer biology have led to the identification of new targets in neoplastic cells and the development of novel targeted therapies. At this time, two targeted agents are approved by the FDA in advanced non-small cell lung cancer (NSCLC): the epidermal growth factor receptor (EGFR) tyrosine kinase inhibitor (TKI) erlotinib, and the anitangiogenic bevacizumab. A third agent, cetuximab, which was recently shown to enhance survival when used with cisplatin and vinorelbine as first line therapy for advanced NSCLC, will likely be approved by regulatory agencies. With more than 500 molecularly targeted agents under development, the prospects of identifying novel therapies that benefit individual patients with lung cancer are bright.

## Introduction

Lung cancer is the leading cause of cancer deaths for both men and women. It accounts for an estimated 15% of all new cancer cases diagnosed in the United States in 2008, and is responsible for an estimated 29% of all cancer deaths [1]. World-wide, the impact of lung cancer is enormous, with 1.35 million cases and approximately 1.18 million deaths [2]. Non-small cell lung cancer (NSCLC), which accounts for approximately 85% of all cases of lung cancer, will cause an estimated 161,840 deaths in the United States in 2008 [1]. Approximately 70% of patients with NSCLC have inoperable locally advanced tumors or metastatic disease at the time of diagnosis.

In the past two decades the median survival has improved disappointingly little. In 1975 the 5-year relative survival rate for all patients with lung cancer was 13%. In the period from 1996 to 2003 the 5-year survival rate increased to only 16% despite the incorporation of modern chemotherapy regimens and great advances in supportive care [1]. Yet, the future for lung cancer is bright. Chemotherapy improves survival when administered postoperatively to patients with stage II and IIIA NSCLC and when administered with radiation in patients with unresectable stage III disease. The median survival for patients with advanced disease in particular has increased with use of improved chemotherapy, targeted therapies and better supportive care. New insights into the pathogenesis of lung cancer are helping to identify more targets for novel therapies. Some of these exciting new agents will be highlighted here.

### Tyrosine Kinase Receptor (RTK) Mechanisms of Disease

Where normal cells require growth factors in their culture medium in order to grow, cancer cells have a greatly reduced dependence on growth factors for their growth and survival. The reason for this inconsistency was uncovered in 1984 when the sequence of the EGF receptor was identified and found to be similar to the *erbB *oncogene. This oncogene was originally discovered in the genome of the avian erythroblastosis virus, a transforming retrovirus that rapidly induces leukemia in red blood cell precursors (erythroleukemia) [3]. The oncoprotein specified by the *erbB *oncogene was found to lack sequences present in the N-terminus of the EGF receptor allowing for constitutive growth and survival signals independent of growth factors that are typically required to activate the normally functioning EGF receptor. Thus, tumor cells, like leukemic cells were not dependent on growth signals for survival.

**Figure 1:**
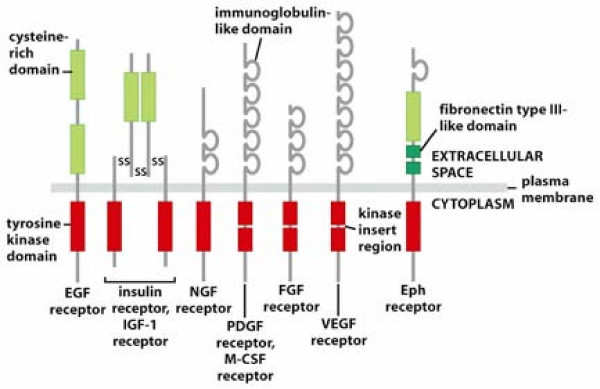
**Tyrosine Kinase Receptor (RTK) families**. Adapted by permission from Macmillan Publishers Ltd: The Biology of Cancer, Garland Science, 2007.

**Figure 2:**
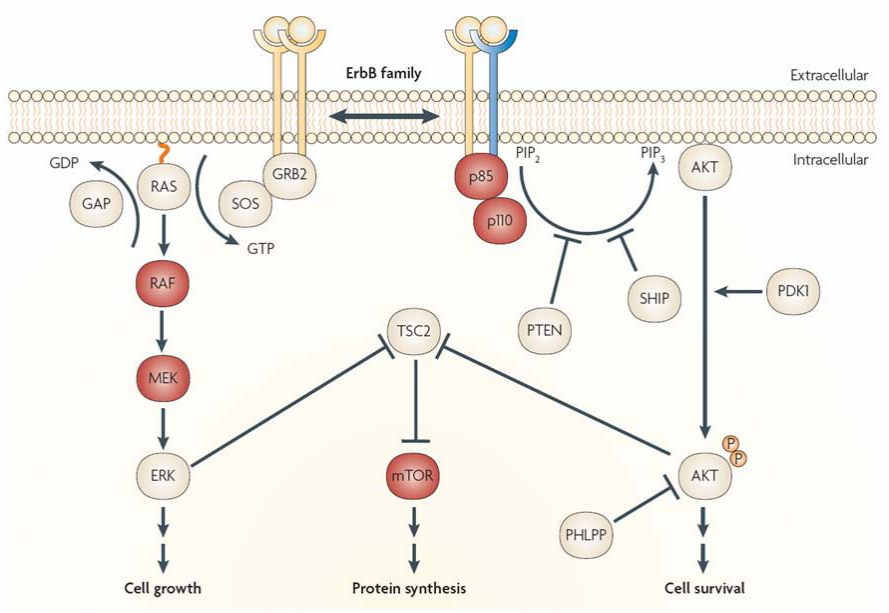
**EGFR signaling pathways**. Two important cell-survival pathways that operate downstream of activated ErbB transmembrane receptor tyrosine kinases (represented by pairs of yellow, and yellow and blue receptors to represent homo- and hetero-dimers, respectively), along with some of the key constituent signaling molecules are shown. The Ras-Raf-MEK-ERK pathway is shown on the left, and the phosphatidylinositol 3-kinase (PI3K)-AKT pathway is shown on the right. Key points along the pathway where targeted inhibition seems to exert a blockade are indicated by red circles, showing the relevant proteins they target. ERK, extracellular signal-regulated kinase; GRB2, growth factor receptor-bound protein 2; mTOR, mammalian target of rapamycin; SOS, son of sevenless. Used with permission from: Nature Reviews 2007 Sharma et al. Pg 177.

The EGF receptor is only one of a large number of similarly structured receptors that contain intracellular tyrosine kinase domains. The unique extracellular domain of these tyrosine kinase receptors (RTKs) is what permits them to be classified into distinct families (Figure 1). When activated by binding specific ligands, RTKs dimerize and phosphorylate the intracellular tyrosine kinase portions of the protein. The activated receptor molecule then may phosphorylate and trigger a diverse array of downstream signaling pathways, including the Ras-Raf-MEK (mitogen-activated and extracellular-signal regulated kinase kinase), ERK1 and ERK2 (extracellular-signal regulated kinase 1 and 2) pathway leading to cell growth, the mTOR (mammalian target of rapamycin) pathway leading to protein synthesis, and the PI3K-AKT (phosphatidylnositol-2 kinase Akt) pathway sustaining cell survival (Figure 2).

In cancer cells, abnormal cell signaling through the RTK pathways is initiated by various mechanisms including: increased production of growth factors, overexpression of growth factor receptors on the cell membrane, and mutations in the receptor or downstream signaling enzymes. The end results are: proliferation, block of apoptosis, angiogenesis, and metastasis [4-6].

### Epidermal Growth Factor Receptor (EGFR)

**Figure 3:**
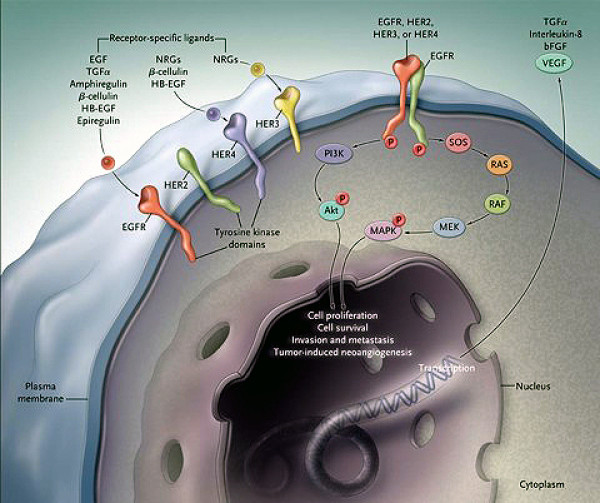
**EGFR signal transduction pathways**. Three steps can be schematically defined in the activation of EGFR-dependent intracellular signaling. First, the binding of a receptor-specific ligand occurs in the extracellular portion of the EGFR or of one of the EGFR-related receptors (HER2, HER3, or HER4). Second, the formation of a functionally active EGFR-EGFR dimer (homodimer) or an EGFR-HER2, EGFR-HER3, or EGFR-HER4 dimer (heterodimer) causes the ATP-dependent phosphorylation of specific tyrosine residues in the EGFR intracellular domain. Third, this phosphorylation triggers a complex program of intracellular signals to the cytoplasm and then to the nucleus. The two major intracellular pathways activated by EGFR are the RAS-RAF-MEK-MAPK pathway, which controls gene transcription, cell-cycle progression from the G1 phase to the S phase, and cell proliferation, and the PI3K-Akt pathway, which activates a cascade of anti-apoptotic and prosurvival signals. bFGF, basic fibroblast growth factor, HB-EGF, heparin-binding EGF, MAPK, mitogen-activated protein kinase, PI3K, phosphatidylinositol 3,4,5-kinase, TGFa transforming growth factor alpha, and VEGF, vascular endothelial growth factor. Used with permission from: NEJM 2008 Ciardiello et al.).

There are 4 members of the EGFR family: EGFR, HER2, HER3, and HER4. Their interactions with extracellular ligands as well as downstream signaling pathways are summarized in Figure 3. After a ligand binds to a single-chain EGFR, the receptor forms a dimer that leads to intracellular phosphorylation and exposure of the catalytic cleft, activating a diverse array of downstream signaling pathways.

There are two classes of EGFR antagonists that are used in clinical practice for non-small cell lung cancer at this time: anti-EGFR monoclonal antibody (cetuximab), and small-molecule EGFR tyrosine kinase inhibitors (TKIs) (gefitinib and erlotinib).

### First Generation Small Molecule TKIs: Gefitinib and Erlotinib

Gefitinib was the first anti-EGFR agent shown to have clinical activity. In two phase II trials gefitinib was evaluated in patients with advanced non-small cell lung cancer, stage III or IV, who were treated with one or more regimens containing cisplatin or carboplatin and docetaxel and had progressed. In both studies symptom improvement rates were around 40%, with 1-year overall survival rates ranging between 25–35% [7,8]. These results, as well as the observation that a few patients had dramatic responses, resulted in approval for gefitinib, prior to a phase III study, as second-line therapy.

The subsequent phase III trial comparing gefitinib with placebo as second line therapy failed to show an improvement in survival. Neither median survival nor the rate of survival at 1 year differed significantly between the two study arms [9]. Pre-planned subgroup analysis showed a significant survival benefit for patients of Asian heritage, and those who never smoked. Based on these results the FDA restricted the use of gefitinib to patients participating in a clinical trial or continuing to benefit from treatment already initiated.

Recently, gefitinib was evaluated in a randomized phase II trial that compared gefitinb with vinorelbine in chemotherapy naïve elderly patients (age > 70 years) with advanced NSCLC. Patients were assigned to gefitinb 250 mg/day orally or vinorelbine 30 mg/m2 infusion on days 1 and 8 of a 21-day cycle. With nearly one hundred patients in each study arm, there was no statistical difference between gefitinb and vinorelbine in efficacy, but there was better tolerability with gefitinib (treatment-related grade 3 to 5 adverse events with gefitinib were 12.8% vs. 41.7% for vinorlebine) [10].

A second small-molecule EGFR tyrosine kinase inhibitor, erlotinib, was also found to have anti-tumor activity in phase II trials [11-13], but, unlike gefitinib, demonstrated improved survival in a placebo controlled phase III study. In the BR.21 trial, treatment with erlotonib was associated with a 2-month increase in survival in previously treated patients with NSCLC. The median overall survival for patients on the placebo group was 4.7 months compared with 6.7 months for the erlotonib group (hazard ratio [HR], 0.70; P < 0.001) [14]. The majority of patients in both arms had a performance status (PS) of 0–1 (68.3% in the placebo group and 65.6% in the erlotinib group). A significant number of patients had a PS of 2, 23% in the placebo group and 25.8% in the erlotinib group. Only 8.6% of patients in both groups had a PS of 3. 50% of patients in erlotinib group as well as the placebo group had previously received one chemotherapy regimen, and half received two or more regimens. In the BR.21 trial the response was higher among Asians, women, patients with adenocarcinoma, and lifetime nonsmokers. Also, the response rate was higher when 10 percent or more of tumor cells expressed EGFR. The presence of EGFR gene mutations was not predictive of a survival benefit from erlotinib. Based on these results, erlotinib was approved for second and third line therapy in NSCLC. The improvement in overall survival seen with erlotinib in the BR.21 trial was comparable to the benefit from docetaxel in the second-line setting [15]. In a separate analysis of BR.21 patients, erlotinib was also shown to improve tumor-related symptoms, physical function (31% erlotinib vs. 19% placebo, P = 0.01), and global quality of life (35% vs. 26%, P < 0.0001) [16].

Four phase III, double-blind, placebo-controlled, randomized clinical trials evaluated erlotonib or gefitinib with chemotherapy as first-line treatment for non-small-cell lung cancer [17-20] (Table 1). Despite the enhanced survival in patients after progression from initial therapy, neither a survival advantage nor a benefit with respect to the response rate or time to progression was seen with the addition of gefitinib or erlotinib to chemotherapy in any of these trials. A retrospective subgroup analysis suggested that the addition of erlotinib to carboplatin and paclitaxel significantly prolonged survival only in the subgroup of patients who had never smoked [19]. Two possible explanations for the lack of benefit when TKIs are added to chemotherapy are interactions between TKIs and chemotherapy and lack of patient selection for the TKI target (EGFR) [21]. TKIs result primarily in G1 cell arrest in cancer cell lines with wild type EGFR, versus induction of apoptosis in cell lines with mutant EGFR [22]. The combination of chemotherapy and TKI in some cases may cause a G1 arrest of growth that blocks the subsequent effects of chemotherapy. In addition, a lack of patient selection for the target (EGFR) may also explain the lack of benefit of TKIs [21,23]. In the phase III TRIBUTE study, for example, that evaluated the efficacy of erlotinib plus carboplatin and paclitaxel versus chemotherapy alone, K-RAS mutations were found in 20% of the patients. These mutations are generally associated with resistance to TKI therapy (see section: The Role of EGFR Mutations in NSCLC). Patients with K-RAS mutations who received erlotinib plus chemotherapy demonstrated worse overall survival (HR = 2.1; 95% CI, 1.1 to 3.8; P = 0.02) than patients who received chemotherapy alone [19]. This is similar to the observation that K-RAS mutations in colon cancer do not benefit from treatment with cetuximab [24-26].

**Table 1:** Selected phase II and III clinical trials of anti-EGFR drugs in non-small cell lung cancer

**Study**	**Disease Setting**	**Treatment (dose) (No. of patients)**	**ORR (CR+PR) (%)**	**mTTP (months)**	**mPFS (months)**	**mOS (months)**
Single arm phase II (Perez-Soler et al.)	Metastatic platinum refractory disease	erlotinib monotherapy (150 mg/day) (57)	12.3	N.R.	N.R.	8.4

Randomized phase II, IDEAL 1 trial (Fukuoka et al.)	Metastatic platinum refractory disease (second and third line of treatment)	gefitinib monotherapy (250 mg/day) (103) gefitinib monotherapy (500 mg/day) (106)	18.4 19.0 (p = NS)	N.R.	2.7 2.8 (p = NS)	7.6 8.0 (p = NS)

Randomized phase II, IDEAL 2 trial (Kris et al.)	Metastatic platinum and Docetaxel refractory disease (third line of treatment)	gefitinib monotherapy (250 mg/day) (102) gefitinib monotherapy (500 mg/day) (114)	12 9 (p = NS)	N.R.	N.R.	7.0 6.0 (p = NS)

Randomized phase III, BR.21 trial (Sheperd et al.)	Metastatic platinum refractory disease (second and third line of treatment)	erlotinib monotherapy (150 mg/day) (448) Placebo (243)	9 <1 (p < 0.0001)	N.R.	2.2 1.8 HR 0.70 (95% CI, 0.58–0.87) (p < 0.001)	6.7 4.7 HR 0.61 (95% CI, 0.51–0.74) (p = 0.001)

Randomized phase III, ISEL trial (Thatcher et al.)	Metastatic platinum refractory disease (second and third line of treatment)	gefitinib monotherapy (250 mg/day) (1129) Placebo (563)	8 1 (p < 0.0001)	N.R.	N.R.	5.6 5.1 HR 0.89 (95% CI, 0.77–1.02) (p = NS)

Randomized phase III, BETA tiial (Hainsworth et al.)	Metastatic, second line therapy	Erlotinib monotherapy (150 mg/day) (313) erlotinib (150 mg/day) + bevacizumab (15 mg/kg) (313)	6.2 6.2 (p = 0.006)	N.R.	1.7 3.4 HR 0.62 (95% CI 0.52–0.75) (p < 0.0001)	9.2 9.3 HR 0.97 (95% CI, 0.80–1.18) (p = NS)

Randomized phase III, INTEREST trial (Kim et al.)	Metastatic platinum refractory disease (second line of treatment)	gefitinib monotherapy (250 mg/day) (733) Docetaxel (733)	9.1 7.6 (p = NS)	N.R.	2.2 2.7 HR 1.04 (95% CI, 0.93–1.18) (p = NS)	7.6 8.0 HR 1.02 (95% CI, 0.90–1.15) (p = NS)

Randomized phase III, TRIBUTE trial (Herbst et al.)	Metastatic, first line treatment	carboplatin + paclitaxel + erlotinib (150 mg/day) (539) carboplatin + paclitaxel + placebo (540)	21.5 19.3 (p = NS)	5.1 4.9 (p = NS)	N.R.	10.6 10.5 (p = NS)

Randomized phase III, TALENT trial (Gatzmeier et al.)	Metastatic, first line treatment	cisplatin + gemcitabine + erlotinib (150 mg/day) (533) cisplatin + gemcitabine + placebo (536)	31.5 29.9 (p = NS)	5.1 4.9 (p = NS)	N.R.	10.0 10.3 (p = NS)

Randomized phase III, INTACT-1 trial (Giaccone et al.)	Metastatic, first line treatment	cisplatin + gemcitabine + gefitinib (250 mg/day) (365) cisplatin + gemcitabine + gefitinib (500 mg/day) (365) cisplatin + gemcitabine + placebo (363)	51.2 50.3 47.2 (p = NS)	N.R.	5.8 5.5 6.0 (p = NS)	9.9 9.9 10.9 (p = NS)

Randomized phase III, INTACT-2 trial (Herbst et al.)	Metastatic, first line treatment	carboplatin + paclitaxel + gefitinib (250 mg/day) (345) cisplatin + paclitaxel + gefitinib (500 mg/day) (347) cisplatin + paclitaxel + placebo (345)	30.4 30 28.7 (p = NS)	N.R.	5.3 4.6 5.0 (p = NS)	9.8 8.7 9.9 (p = NS)

NSCLC, non-small-cell lung cancer; ORR, overall response rate; CR, Complete response; PR, partial response; mPFS, median progression free survival; m TTP, median time to progression; mOS: median overall survival; HR, hazard ratio; CI, confidence interval; N.R.: not reported.

Dose-dependent and reversible diarrhea and acne-like rashes are the most frequently reported side effects of TKIs. The histologic characteristics of the rash include a neutrophilic infiltrate in perifollicular areas within the basal layer of the skin [19,27].

### Monoclonal Antibodies Against EGFR: Cetuximab, Panitumumab, and Matuzumab

Monoclonal antibodies that bind the extracellular domain of EGFR prevent the receptor from interacting with its ligand, EGF, and thus prevent intracellular signal transduction. In addition, antibodies have the inherent ability to recruit immune effector cells such as macrophages and monocytes to the tumor through the binding of the antibody constant Fc domain to specific receptors on these cells. This immune mechanism has been demonstrated in xenograft models [28]. Cetuximab is a human-mouse chimeric monoclonal antibody (IgG1 subtype) that demonstrated activity in NSCLC. In phase 2 studies, where cetuximab was added to platinum-based regimens, clinical benefit was reported [29-33]. In the phase III FLEX trial where cetuximab with cisplatin/vinorelbine was compared with ciplatin/vinorelbine alone in 1,125 patients with EGFR-detectable advanced NSCLC, a statistically significant improvement in overall survival for the cetuximab group was reported (11.3 months vs. 10.1 months HR 0.871; 95% CI, 0.762–0.996; P = 0.0441). The median age of patients in both study arms was 59 years, and 94% of patients had stage IV disease [34]. Based on this large phase III trial, the current recommendations from the National Comprehensive Cancer Network, Inc. (NCCN) include cetuximab/vinorelbine/cisplatin as a first-line therapy option in patients who meet criteria for therapy with cetuximab (i.e. NSCLC IIIB with a pleural effusion or stage IV, EGFR expression by immunohistochemistry [≥ 1 positive tumor cell], age ≥ 18, ECOG PS 0–2, no known brain metastasis and no prior chemotherapy or anti-EGFR therapy) [35]. Data on the role of K-RAS mutations as predictive for benefit from cetuximab in NSCLC is expected.

Cetuximab is relatively well tolerated. The most common adverse events reported in a phase I trial were fever and chills, asthenia, skin toxicity (flushing, acne-like rash, and folliculitis), transient elevations in aminotransferase levels, and nausea [36].

Panitumumab (ABX-EGF, Vectibix^®^), a fully human monoclonal antibody (IgG2k subtype), and matuzumab (EMD 72000), a humanized monoclonal antibody (IgG1 subtype) are in phase II and III testing. Both target EGFR but at different epitopes. Panitumumab binds domain III of EGFR, the same locus as cetuximab, and thus blocks all known EGFR ligands. This results in inhibition of receptor activation [37]. Matuzumab binds to a distinct portion of domain III, and unlike panitumumab and cetuximab, sterically blocks the domain rearrangement that is required for high-affinity ligand binding and receptor dimerization [38].

Panitumumab was well tolerated in phase I studies, where the most common toxicity was a transient acneiform skin rash, typically grade 1 or 2. No human antihuman antibodies have been reported to date [39,40]. A randomized phase II trial in previously untreated advanced stage IIIB and stage IV NSCLC patients compared carboplatin (AUC 6 IV every 3 weeks) and paclitaxel (200 mg/m2 IV every 3 weeks) with or without panitumumab (2.5 mg/kg weekly). In this trial there was no benefit appreciated with regard to time to disease progression (4.2 vs. 5.3 months for chemotherapy alone, P = 0.55). Also, there was no reported benefit in response rate or median survival time. Based on this disappointing phase II trial there has been little enthusiasm for evaluating panitumumab in a phase III trial [40,41]. Nevertheless, this situation requires reassessment in view of the positive trial with cetuximab.

Matuzumab, another monoclonal antibody that targets EGFR is approximately 90% humanized and 10% murine. In phase I testing it was well tolerated with grade 1 or 2 skin toxicity reported in two thirds of the patients [42,43]. It has a half-life of approximately 10 days permitting effective administration once every two or three weeks [44]. Matuzumab is currently undergoing phase II evaluation in NSCLC [45].

### Predictors of Response-The Role of EGFR Mutations in NSCLC

Predicting which patients are most likely to benefit from EGFR targeted therapy remains a challenge. The studies of erlotinib and gefitinib identified a population that is more likely to respond to anti-EGFR therapy, i.e. never-smokers, of Asian heritage, female sex, and a tumor with adenocarcinoma histology. The presence of cutaneous side effects has also been correlated with response rates [46].

At the molecular level, most patients with partial or complete responses to gefitinib and erlotinib harbored specific mutations in the gene that encodes EGFR, located on chromosome 7p12 [47]. Exon 19 mutations, characterized by in-frame deletions of amino-acids 747–750, account for 45% of mutations, exon 21 mutations, resulting in L858R substitutions, account for 40–45% of mutations, and the remaining 10% of mutations involve exon 18 and 20 [48-51]. These mutations have been shown, in vitro, to increase the kinase activity of EGFR, leading to the hyperactivation of downstream pro-survival pathways, and consequently confer oncogenic properties on EGFR [52-54]. These mutants are also more sensitive to inhibition by gefitinib and erlotinib than are the wild-type receptors.

Overall, the incidence of EGFR mutations in NSCLC among clinical responders to gefitinb or erlotinib is 77%, compared with 7% in NSCLC cases that do not have a CR or PR [55-57]. In studies with unselected NSCLC patients, EGFR mutations are found in approximately 10% of cases in North America and Western Europe, and approximately 30–50% of cases from East Asia [49,50]. These mutations may be limited to non-small-cell lung cancer, as they are rarely identified in other human cancers. The presence of EGFR kinase mutations seem to be highly correlated with clinical characteristics, i.e. female sex, never smokers, Asian descent, adenocarcinoma histology, whereas, in patients with smoking-associated cancers, EGFR gene amplification, as measured by qPCR may be an oncogenic driving force [58].

Increased EGFR gene copy number as determined by fluorescent in situ hybridization (FISH) and EGFR protein overexpression measured by immunohistochemistry (IHC) are correlated with improved response and survival to TKI therapy [59,60]. In the BR.21 trial, for example, the positive treatment effect of erlotinib was confined to the EGFR FISH positive patients (gene amplification and/or high polysomy) both in terms of response rate (20% for FISH positive and 2% for FISH negative) and survival (HR, 0.44 for FISH positive and HR, 0.85 for FISH negative) [61]. However, in a multivariable analysis no molecular markers were predictive for survival.

In a cohort of NSCLC patients from Italy treated with gefitinib, EGFR protein overexpression (IHC positive) was demonstrated in 59% of tumors, and was associated with increased response (21% vs. 5%; P = 0.03) and survival (11.5 vs. 5 months; P = 0.01), but not with specific clinical characteristics. The majority of mutation positive cases that responded to treatment were also FISH positive; however, both IHC positive status and EGFR mutations were associated with FISH positivity [59,62].

In the ISEL trial evaluating gefitinib in NSCLC, the subgroup of patients with EGFR mutations had a higher response rate to TKI therapy. Twelve percent of patients were found to have EGFR mutations, and they had a higher response rate (37.5%) with gefitinib treatment than mutation-negative patients (2.6%, P value not reported). FISH positive status was observed in 30.8% of patients and was associated with a nonsignificant trend toward improved survival with gefitinib treatment (HR = 0.61; 95% CI, 0.36 to 1.04) [63].

The INVITE trial, that compared gefitinb with vinorelbine in chemotherapy naïve, unselected elderly patients with advanced NSCLC, reported no statistical difference in outcome, with improved tolerability for gefitinib. One unexpected finding was noted in the EGFR-FISH analysis: individuals who were FISH positive appeared to benefit to a greater extent from vinorelbine than from gefitinib. This finding was in contrast with previous trials that showed a survival improvement for patients who were EGFR FISH-positive and who received an EGFR-TKI. A sampling error due to incomplete EGFR FISH testing may have contributed to these findings. For example, the authors reported that this analysis was limited in that mutation analysis was performed in a "limited number of instances," because ethics committee approval was obtained in only a few centers [10].

Preliminary results from the IPASS study were presented at the European Society for Medical Oncology in September of 2008. This phase III trial evaluated gefitinib vs. carboplatin/paclitaxel in 1217 Asian patients with advanced NSCLC who had not received prior systemic therapy and who had never smoked or were light former smokers. Based on clinical factors the population was enriched for EGFR mutations. Indeed, among the evaluable patients, the overall EGFR mutation positive rate was 59.7%. The primary endpoint was progression free survival (PFS), and it showed a significant difference favoring gefitinib (HR = 0.68; 95% CI, 0.58 to 0.81; P < 0.0001). Among patients with EGFR mutations the response rate was significantly greater for those treated with gefitinib (odds ratio [OR] 2.75; 95% CI, 1.65 to 4.6, P = 0.001) while in patients without an EGFR mutation response rate was greater with chemotherapy (OR 0.04; 95% CI, 0.01 to 0.27; P = 0.0013). Quality of life analysis favored gefitinib as well (P = 0.0148). Median overall survival appeared similar between the two groups although definitive results were not presented [64]. An update presented at the Chicago Multidisciplinary Symposium in Thoracic Oncology in November 2008 verified the earlier findings, and reported improved quality of life scores for patients receiving gefitinib compared with chemotherapy. Likewise, gefitinib had a more favorable tolerability profile than carboplatin/paclitaxel [65]. This trial supports the observation that patients with EGFR mutations have a better prognosis and may benefit from both TKI therapy and from cytotoxic chemotherapy.

The INTEREST trial was a randomized phase III trial that compared gefitinib versus docetaxel in previously treated NSCLC. In this trial, the patients were randomly assigned after dynamic balancing with respect to histology (adenocarcinoma vs. other). The authors reported that specific clinical factors (never-smokers, Asian origin, female sex, and adenocarcinoma histology) were associated with a longer survival in both the gefitinib and docetaxel groups [66]. This was unexpected since previous trials suggested that chemotherapy produces similar survival in all patients.

Another trial evaluated EGFR mRNA expression and gene dosage, both assayed by quantitative PCR (qPCR) in tumor samples from patients with gefitinib-treated NSCLC. Unlike FISH that allows for quantification of gene copy number in individual tumor cells, qPCR techniques assess gene copy number or mRNA levels in a pool of cells. Often tumor microdissection is necessary to ensure that a high percentage of tumor cells are present in the analyzed sample. Also, deletions or amplifications of genetic material within tumor cells may limit the accuracy of qPCR [67]. In this trial, EGFR mRNA expression was predictive of response to gefitinib therapy and for PFS after treatment, while EGFR gene dosage was not associated with a response to therapy or outcome. Also, high EGFR mRNA expression was correlated with increased EGFR gene copy number as evaluated by FISH [68]. These findings support the use of qPCR to determine EGFR mRNA expression in NSCLC.

One of the downstream messengers of EGFR that transduces the EGFR activation signal within the cell is K-RAS. K-RAS gene mutations on codons 12, 13, and 61 result in constitutive activation of the RAS protein, which may render tumor cells independent of EGFR signaling and also resistant to anti EGFR therapy [69]. Significantly, K-RAS mutations are found almost exclusively in smoking-associated NSCLC with wild-type EGFR [70-72].

In the previously described phase III TRIBUTE trial that compared chemotherapy with carboplatin/paclitaxel alone to the same regimen with the addition of erlotinib, patients with K-RAS mutations in the erlotinib group had a worse survival than those who received chemotherapy alone [19,73]. A similar retrospective analysis was performed in patients on the BR.21 trial. In this trial, 10% of 98 K-RAS wild-type patients assessable for response had confirmed response to erlotinib, whereas only one of the 20 K-RAS mutant patients responded (this patient also had EGFR amplification) [74]. Genetic analysis of both trials supports the theory that NSCLC patients with K-RAS mutations are unlikely to respond to anti EGFR therapy.

Another subgroup analysis from the TRIBUTE study evaluated EGFR gene copy number using FISH found that the EGFR gene copy number did not predict an overall survival benefit. However, among EGFR FISH positive patients the time to progression was longer in patients who received erlotinib and continued to receive it after completing first-line therapy (HR = 0.59; 95% CI, 0.35 to 0.99; P = 0.0403) [75]. This lends additional support to the lack of benefit of combining chemotherapy with TKIs, while suggesting the possible benefit of TKI therapy as part of a maintenance regimen. The point where the TTP curves diverged was after 6 months, when erlotinib was continued alone. The ATLAS trial of maintenance bevacizumab +/- erlotinib may help clarify the utility of TKIs in maintenance therapy for NSCLC. The trial is now closed, and results are expected in the first half of 2009 [76,77].

### Acquired Resistance to EGFR-Targeted Therapy

In approximately 50% of patients who initially respond to TKIs but later relapse, the T790M mutation in exon 20 of the EGFR gene occurs as a single secondary event [78,79]. It has been proposed that this second mutation may weaken the interaction of inhibitors with the target kinase [80]. Other possible routes for acquired resistance to TKIs include: metalloproteinase 17 (ADAM17) mediated autocrine activation of ERBB2 and ERBB3, amplification of EGFR, hyperactivation of downstream signaling components that circumvent EGFR inhibition, cellular changes that alter the bioavailability of the inhibiting drugs, and drug-resistance through ATP-binding cassette GE (ABCG2) transporter which actively pumps the cytotoxic agent out of the tumor cells [48,81].

### Second Generation Small Molecule TKIs

Novel agents have been designed to overcome the steric interference to drug binding that is conferred by the T790M and other mutations. One group of drugs that bind irreversibly to the active site of EGFR was shown in vivo to overcome the resistance to EGFR RTKs. These have been termed second generation TKIs. A summary of the early studies involving these agents is included in Table 2[82-87]. One example among the second generation TKIs is XL647. This is a reversible inhibitor of EGFR, HER2, and vascular epidermal growth factor receptor (VEGF). Preclinical evaluation demonstrates that XL647 can inhibit cell lines bearing mutated forms of EGFR that have been associated with acquired resistance [82,84]. Preliminary data from phase II trial showed a response rate of 29% (N = 34). In patients with tissue available, EGFR mutation analysis was performed. Although 6 of the 10 patients with partial response had EGFR mutations, 3 patients had wild-type EGFR. Of the seven patients with classic EGFR mutations, six had a partial response, and one had prolonged stable disease [85].

**Table 2:** Targeted therapeutic agents in NSCLC

**Class**	**Agent**	**Target**	**Company**	**Stage of development in NSCLC**
First Generation TKI				

	Gefitinib	EGFR (reversable)	AstraZeneca	Approved for a restricted group of patients

	Erlotinib	EGFR (reversable)	OSI, Genentec and Roche	Approved

Second Generation TKI				

	EKB-569	EGFR (irreversible)	Wyeth	Phase II

	CL-387,785	EGFR (irreversible)	Wyeth	Preclinical

Multi-Targeted TKI				

	HKI-272	EGFR, HER2 (irreversible)	Wyeth	Phase I/II

	Canertinib	EGFR, HER2, HER4 (irreversible)	Pfizer Inc.	Phsae II

	BIBW 2992	EGFR, HER2 (irreversible)	Boehringer Ingelheim	Phase I/II

	HKI-357	EGFR, HER2 (irreversible)	Wyeth	Preclinical

	Vandetanib, ZD-6474	EGFR, HER2, FLT1, KDR (reversible)	AtraZeneca	Phase III

	XL647	EGFR, HER2, KDR, EPHB4 (reversible)	Exelexis	Phase II

HER2 Heterodimerization				

	BMS-599626	EGFR, HER2	Bristol-Myers Squibb	Phase I

Macrolide Derivatives				

	RAD001	mTOR	Novartis Pharma AG	Phase II

	CCI-779	mTOR	Wyeth	Phase II

	AP23573	mTOR	Ariad Pharmaceuticals	Phase I

Monoclonal Antibodies				

	Cetuximab	EGFR (chimeric mAB)	ImClone/Merk KGaA Bristol-Myers Squibb	Approved

	Matuzumab	EGFR (humanized mAb)	Merck KgaA	Phase II

	Panitumumab	EGFR (humanized mAb)	Abgenix	Phase II/III,

	Trastuzumab	HER2 (humanized mAb)	Genentech/Roche	Approved

	Bevacizumab	VEGF-A	Genentech	Approved

VEGF Inhibitors				

	Sorafenib	VEGFR2, FLT3, PDGFR, fibroblast growth factor receptor-1	Bayer HealthCare Pharmaceuticals and Onyx Pharmaceuticals	Phase III

	Sunitinib	c-kit, VEGFR1-3, PDGFRa, PDGFRb, Flt-3, CSF-1R, ret	Pfizer Inc.	Phase II/III

	Axitinib AG013736	VEGF 1-3, PDGFR, cKIT	Pfizer Inc.	phase II

	Regeneron	VEGF-Trap		Phase I

Non VEGF Angiogenesis inhibitors				

	Celecoxib	COX-2	Pfizer Inc.	Phase II

Proteasome Inhibitors				

	Bortezomib	Inhibits 26S proteasome	Millennium Pharmaceuticals, Inc.	Phase II

Retinoic Acid Receptor				

	Bexarotene	Retinoid × receptor	Eisai Inc.	Phase III

The most common therapy related adverse events for XL647 were grade 1 or 2 diarrhea, rash, fatigue and nausea. Phase II data revealed that nearly 50% of patients experienced a prolongation in the QTc. The vast majority of these EKG changes were grade 1 or 2, although 6% of patients were found to have grade 3 toxicity [85].

### Targeting HER2 in NSCLC

HER2 is a member of the EGF (ERBB) family of tyrosine kinase receptors to which EGFR also belongs. HER2 is dysregulated in many cancers, where it is commonly overexpressed by amplification. When HER2 is overexpressed, as in breast and ovarian cancers, it is associated with a poor prognosis [88,89].

Signal transduction by HER2 is distinct from other members of the EGF family of receptors. For example, the binding of EGFR to it's ligand induces the formation of homo and hetero-dimers among the EGFR related receptors. Dimerization results in activation of the intrinsic kinase domain within the cell. This contrasts with HER2 activation that (unlike EGFR, HER3, and HER4) does not have an extracellular ligand-binding site (receptor). It dimerizes with other members of the EGF family (heterodimer) or with itself (homodimer). The strongest and the most potent heterodimer formed is EGFR/HER2 [90].

Recent studies have reported that mutations in the tyrosine kinase domain of HER2 are occasionally detected in lung cancers [91]. One retrospective trial, for example, analyzed tumors from 116 patients in relation to smoking status. EGFR mutations were detected in 20 of 116 (17%) tumors, whereas five (4.3%) tumors contained HER2 mutations. No tumor contained both mutations. Of tumors with EGFR or HER2 mutation, 72% were adenocarcinomas, 68% were from never smokers, and 32% were from former smokers. EGFR but not HER2 mutations were mutually exclusive with KRAS mutation [89].

This small study highlights the diversity of genetic aberrations identified in NSCLC. Some of the second generation TKIs that target HER2 along with EGFR may show activity in patients who initially respond to TKIs but later develop resistance, if that resistance is mediated by mutations in HER2.

Trastuzumab, a monoclonal antibody directed against HER2, has been evaluated in NSCLC. It had no significant clinical activity when given either as a single agent or in combination with platinum based chemotherapy even in NSCLC with over expression of HER2 [92-96]. A pan HER inhibitor, PF-00299804, that binds irreversibly to EGFR, HER2, and HER4, in a phase I trial induced 2 PRs among 44 patients with advanced NSCLC after failure of prior treatment with reversible EGFR inhibitors [97].

### mTOR Inhibitors, Rapamycin Derivatives: CCI-779 (Temsirolimus), RAD001 (Everolimus)

Mammalian target of rapamycin (mTOR) kinase is an important mediator of tumor cell growth and proliferation. It is activated in >50% of lung carcinomas [98]. It is located downstream, along the PI3K-AKT pathway where it serves as a central sensor for nutrient/energy availability [6,99]. In the presence of stimulation at the EGFR receptor in combination with sufficient nutrients and energy, the mTOR pathway is activated, and cell growth is initiated.

Several agents that inhibit mTOR are currently in clinical trials. Preliminary results from the first 50 patients enrolled in a phase II trial of CCI-779 who were previously untreated for NSCLC reported 4 patients with a partial response (PR rate of 8%), and 15 patients with stable disease (SD rate of 30%). The median PFS time was 2.3 months and the median OS time was 6.6 months (100,101). The most common grade 3 or 4 toxicities for CCI-779 were dyspnea (12%), fatigue (10%), hyperglycemia (8%), hypoxia (8%), nausea (8%), and rash (6%).

Another mTOR inhibitor, RAD001 was evaluated in a phase II of patients with an ECOG performance status of two or higher who failed ≤ 2 cycles of platinum-based therapy (arm 1) vs. those who failed ≤ 2 cycles of platinum-based therapy as well as an EGFR antagonist. From 74 evaluable patients, the median PFS was 11.3 weeks in arm 1 and 9.7 weeks in arm 2. The most frequent adverse events were stomatitis/mucositis, cough, dyspnea, rash, fatigue, anorexia, nausea, anemia, epistaxis and diarrhea. The molecular marker portion of the study is still ongoing [102].

An exciting phase II trial is currently underway combining mTOR and EGFR inhibition in NSCLC. There is some preclinical data suggesting synergy between gefitinib and everolimus [103]. This regimen was tolerable for patients in phase I trials, although the incidence of diarrhea, rash and mucosal ulcerations were high [104-106].

### Targeting Angiogenesis and VEGF

**Figure 4:**
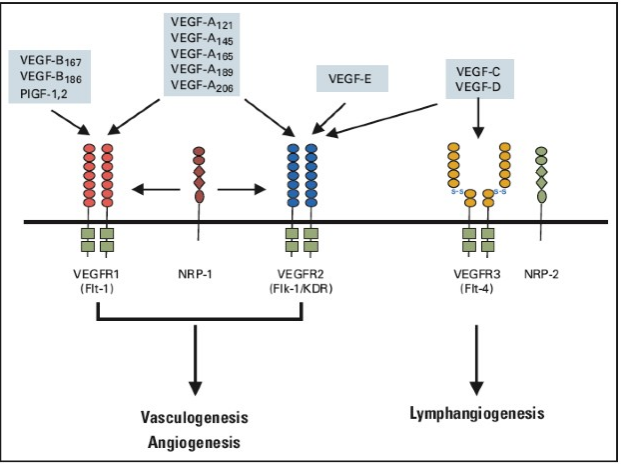
**VEGF signaling pathways**. Binding specificity of various vascular endothelial growth factor (VEGF) family members and their receptors. The VEGF family consists of seven ligands derived from distinct genes (VEGF-A, -B, -C, -D, and -E, placenta growth factor [PIGF] -1 and -2). VEGF family members have specific binding affinities to VEGF receptor (VEGFR) -1, VEGFR-2 and BEGFR-3 tyrosine kinase receptors as shown. In addition, neuropilin (NRP)-1 and NRP-2 are co-receptors for specific isoforms of VEGF family members and increase binding affinity of these ligands to their respective receptors. Used with permission from: Hicklin DJ, Ellis LM. Role of the vascular endothelial growth factor pathway in tumor growth and angiogenesis. J Clin Oncol 2005; 23: 1011–27.

Like normal tissue, tumors require access to the circulation in order to grow and survive. The process of developing vasculature through angiogenesis is complex, and offers multiple diverse targets for anti-cancer therapeutics. Vascular endothelial growth factor is the dominant growth factor controlling angiogenesis. VEGF comprises a family of growth factors including: placental growth factor, VEGF-A, VEGF-B, VEGF-C, VEGF-D, and VEGF-E (orf virus VEGF) (Figure 4).

VEGF-A is the major mediator of tumor angiogenesis, and is the target of the monoclonal antibody bevacizumab [107-109]. VEGF ligands mediate angiogenesis via several receptors including VEGFR-1 (Flt-1) and VEGFR-2 (KDR, Flk-1), and lymphangiogenesis via VEGFR-3 (Flt-4) [109-111]. Normal endothelial cells express VEGFR-2, and normal vascular tissues express either VEGFR-1 or VEGFR-3. This contrasts with tumors that have been shown to express several different VEGF ligands simultaneously [109,112]. VEGF receptors in normal tissues are involved in a range of cellular pathways that vary with the stage of development of the organism as well as with the physiologic and pathologic conditions. Both VEGFR-1 and VEGFR-2 can bind the VEGF-A ligand promoting angiogenesis. VEGFR-1 is critical for physiologic and developmental angiogenesis [113,114]. VEGFR-2 mediates microvascular permeability, endothelial cell proliferation, invasion, migration, and survival. Signaling by VEGF-2 may be positively or negatively influenced by co-expression and activation of VEGFR-1.

In growing tumors VEGFR-1 and VEGFR-2 have been shown to be a potent positive regulator of angiogenesis [113]. VEGFRs have been identified on the surface of tumor cells in a range of malignancies including NSCLC [114]. It has been proposed that tumor cells abnormally expressing VEGFRs that also secrete VEGF induce an autocrine loop promoting tumor angiogenesis [115]. Support of this hypothesis is demonstrated by activation of MAPK pathway in tumor cells after VEGFR-1 activation by VEGF-A or VEGF-B [116,117].

It logically follows that targeting VEGF and VEGFR should destroy the tumor vasculature and starve the tumor of oxygen and nutrients. In fact, VEGF blockade as monotherapy has been clearly shown to have a direct and rapid anti-vascular effect in both animal and human tumors [118]. However, it has also been proposed that certain antiangiogenic agents can also transiently "normalize" the abnormal structure and function of tumor vasculature to make it more efficient for oxygen and drug delivery [119,120]. This supports the use of angiogenesis medication in combination with chemotherapeutic agents.

### Angiogenesis Inhibitors: Bevacizumab

Bevacizumab is a humanized monoclonal antibody directed against VEGF that recognizes all isoforms of VEGF-A. It has a long half life of 17 to 21 days after IV infusion [121]. A pivotal phase III trial in NSCLC, ECOG 4599, showed that adding bevacizumab to paclitaxel plus carboplatin resulted in a survival advantage compared with chemotherapy alone in patients with recurrent or advanced NSCLC. The median survival was 12.3 months in the chemotherapy plus bevacizumab group compared with 10.3 months in the chemotherapy alone group (hazard ratio for death, 0.79; p = 0.003) [122]. In this trial patients with squamous-cell tumors, brain metastasis, clinically significant hemoptysis, or inadequate organ function or performance status (ECOG performance status, >1) were excluded.

The addition of bevacizumab resulted in increased rates of hypertension, proteinuria, bleeding, neutropenia, febrile neutropenia, thrombicytopenai, hyponatremia, rash, and headache when compared with the paclitaxel/carboplatin alone group (P < 0.05). Of significant note was the increased rate of death from pulmonary hemorrhage, cerebrovascular events, and gastrointestinal hemorrhage [122].

Another phase III trial, AVAIL (BO17704), evaluated the addition of bevacizumab to cisplatin/gemcitabine, a regimen that is commonly used in regions outside of the US. This randomized, placebo-controlled phase III study compared two doses of bevacizumab plus cisplatin/gemcitabine to cisplatin/gemcitabine plus placebo in 1,043 patients. The eligibility criteria included: previously untreated advanced or recurrent non-squamous NSCLC, ECOG PS 0–1, and no brain metastases. PFS was significantly prolonged as analyzed both in a primary analysis (without censoring for non-protocol anti-neoplastic therapy [NPT] prior to progression) and in a pre-specified analysis with censoring for NPT. The response rate (RR) and response duration were also increased. An initial company press release indicated that the difference in survival was not statistically significant [123]. The authors concluded that bevacizumab significantly improved PFS and RR, consistent with the results of the earlier phase III trial E4599 [124]. With longer follow-up, the preliminary findings were supported. The risk of progression or death was reduced by 25% with bevacizumab 7.5 mg/kg and 15% with bevacizumab 15 mg/kg vs. placebo (P = 0.003 and 0.046, respectively) [125].

### Angiogensis Inhibitors: AVE0005 (VEGF Trap)

VEGF Trap is a recombinant fusion molecule with a high-affinity for binding to all isoforms of VEGF and to placental growth factor. It has been postulated that the improved affinity may allow more efficient depletion of tissue and plasma VEGF [126]. Initial phase II results in patients with platinum and erlotinib resistant adenocarcinoma of the lung revealed two PRs (6%) and 63% with SD among the first 33 evaluable patients. Grade 3–4 treatment related adverse events included dyspnea (15%), hypertension/non-cardiac chest pain (9%), fatigue (6%), and anxiety, epistaxis, nausea, bone pain, proteinuris, febrile neutropenia, pneumonia, pulmonary emvolism and renal pain (each 3%). No grade 3 or greater hemoptysis was reported [127,128].

### Angiogenesis Inhibitors: COX-2 Inhibitors

Cyclooxygenase-2 (COX-2) is an enzyme in the arachidonic acid cascade that is unregulated and overexpressed in many tumors, including lung cancer. It has been proposed that increased COX-2 enzyme may create a surplus of prostaglandin E2 (PGE2). PGE2 then promotes tumor growth and invasion through the stimulation of VEGF and the upregulation of bcl-2 and various matrix metalloproteinases [129]. In clinical trials COX-2 inhibition with celecoxib has not been shown to be effective when combined with irinotecan/docetaxel or irinotecan/gemcitabine [130].

### Multitargeted Agents: Sunitinib, Sorafenib, Vandetanib and Axitinib

Sunitinib malate is an oral, multitargeted tyrosine kinase inhibitor with antiangiogenic and antitumor activities. It inhibits VEGFR-1, VEGFR-2, VEGFR-3, PDGFR-alpha, PDGFR-beta, KIT, RET and FLT3. In NSCLC it was evaluated in a Phase II clinical trial where 63 patients with advanced NSCLC who failed platinum-based chemotherapy were treated with sunitinib (50 mg/day) for 4 weeks followed by 2 weeks of no treatment for each 6 week cycle. Seven patients achieved a PR, and 18 patients had stable disease. The median progression-free survival was 12.0 weeks (95% CI, 10–16.1 weeks), and the median overall survival was 23.4 weeks (95% CI, 17–28.3 weeks). The 1-year survival rate was 20.2% [131].

The toxicities reported in this trial from sunitinib were predominantly grade 1 to 2, and did not interfere with scheduled treatment. Grade 3 or 4 adverse events included fatigue/asthenia (29%), pain/myalgia (17%), dyspnea (11%), and nausea/vomiting (10%). Three hemorrhage-related deaths were reported among the 63 total participants. Two of the hemorrhage-related deaths were attributed to sunitinib, and both resulted in pulmonary hemorrhage [131].

A second phase II trial with the same inclusion criteria was designed to evaluate a continuous dosing schedule for suntinib. In this trial sunitinib was given 37.5 mg/day orally. 47 patients were accrued and evaluated with a median duration of therapy of 92 days (range 12–336 days). A response rate of 2.1% (95% CI, 0.1 to 11.1) with a 19.1% rate of disease stabilization was reported. The median time to progression was 12.3 weeks (95% CI, 8.9 to 16 weeks), and the median survival time was 38.1 weeks (95% CI, 31.1 to unavailable) [132]. Although the trials cannot be directly compared since they were performed in a sequential fashion, both dosing schedules showed activity in NSCLC.

There are several ongoing clinical studies in NSCLC incorporating sunitinib. One is Cancer and Leukemia Group B (CALGB) 30607 evaluating the use of maintenance sunitinib compared with placebo in patients with advanced stage IIIB or stage IV NCSLC who have non-progressing disease after four cycles of platinum-based chemotherapy. The primary end point is progression-free survival [133]. There is a phase II and a phase III trial underway evaluating the combination of erlotinib with or without sunitinib. In addition, the combinations of sunitinib with other chemotherapeutic agents including docetaxel, platinum, gemcitabine, and pemetrexed are currently underway [133]. A phase I trial presented at the 2007 ASCO annual meeting incorporating sunitinib with docetaxel in patients with advanced solid tumors including 13 patients with NSCLC, showed encouraging results [134].

Sorafenib is an oral multi-kinase inhibitor that targets RAF, VEGFR-2, and VEGFR-3. In a phase II trial that evaluated 54 patients with relapsed or refractory NSCLC approximately 60% of patient achieved disease stabilization [135]. When sorafenib was combined with carboplatin and paclitaxel in 15 patients with advanced, progressive NSCLC the disease control rate (objective response plus stable disease) was 79%. The duration of response was 25 weeks, and the median progression free survival was 34 weeks [136].

One small phase II trial employed sorafenib alone in 25 patients with chemo-naïve stage IIIB (wet) or stage IV patients. Three patients had a PR and 7 patients had stable disease. The PFS and MS was 2.9 and 8.8 months respectively [137].

The phase III ESCAPE trial that evaluated sorafenib with carboplatin/paclitaxel in patients with NSCLC was stopped early when a planned interim analysis concluded that the study would not meet its primary endpoint of improved overall survival. A higher mortality was observed in the subset of patients with squamous cell carcinoma who received sorafenib and chemotherapy compared with those that only received chemotherapy. Another phase III trial with sarafenib, NexUS, is accruing patients [138].

Vandetanib (ZD6474) is a once-daily inhibitor of VEGFR-2 and RET kinase inhibitor. In a phase II trial of patients with locally advanced or metastatic NSCLC who failed first-line platinum-based chemotherapy, vandetanib plus docetaxel demonstrated a significant prolongation of PFS compared with docetaxel, 18.7 vs. 12 weeks (HR = 0.64; one sided P = 0.037) [139]. Based on these encouraging findings, phase III studies of vandetanib are currently underway.

Axitinib (AG-013736) is a small molecule inhibitor that targets VEGFR-1, VEGFR-2, VEGFR-3, PDGFR-beta, and cKIT. It was evaluated in a phase II trial of 32 patients with advanced NSCLC. 72% had received prior chemotherapy. In this trial 3 patients responded. The median duration of response was 9.4 months. The median survival was 12.8 months (95% CI, 9.9 to undefined), and progression-free survival was 5.8 months (95% CI, 3.8 to 10.2 months) [140].

### Targeting the Proteasome: bortezomib

Bortezomib is a proteasome inhibitor that disrupts the ubiquitin-proteasome pathway leading to apoptosis. In a phase II trial of bortezomib alone and in combination with docetaxel in 155 previously treated patients with advanced non-small-cell lung cancer the one-year overall survival was modestly improved in the combined therapy arm, 39% vs. 33% [141]. The most common adverse effects of bortezomib include peripheral neuropathy, transient thrombocytopenia, and gastrointestinal disorders (nausea, diarrhea, and constipation).

A Southwest Oncology Group phase II study (S0339) evaluated 114 patients with chemotherapy naïve wet stage IIIB and stage IV disease. Patients received gemcitabine/carboplatin with bortezomib. Responses were seen in 20% of patients and 45% had stable disease. The overall disease control rate was 66%. PFS and median overall survival were 5 and 11 months, respectively [142]. Based on this trial, a phase III trial is underway [143].

### Targeting the Retinoic Acid Receptor: Bexarotene

Analogues of vitamin A, retinoids, are required for normal growth and differentiation of human bronchial epithelium. When certain retinoid receptors in the cell nucleus such as RAR-beta (retinoic acid receptors) and RXRs (retinoid × receptors) are inactivated, tumors in the lungs may develop [144,145]. In this way, retinoic acid receptors act like tumor suppressors. Bexarotene is a selective retinoid × receptor (RXR) modulator that binds RXR alpha, beta, and gamma. In two phase III trials of bexarotene with either cisplatin/vinorelbine (SPIRIT I) or carboplatin/paclitaxel (SPIRIT II) the addition of the selective retinoic acid receptor inhibitor to chemotherapy did not improve survival. However, it was reported in both studies that the subset of patients who developed hypertriglyceridemia had a significant improvement in median survival compared with controls (12.4 vs. 9.2 months log-rank, P = 0.014; SPIRIT II) [146,147]. The benefit is most pronounced when the hypertriglyceridemia is high-grade and develops rapidly, in patients with the following characteristics: men, stage IV disease, smokers, and those with ≥ 5% weight loss in previous 6 months [147].

## Summary and conclusion

The search for innovative therapeutic agents in NSCLC that are more effective and have fewer side effects than older chemotherapeutic drugs has spurred the development of more than 500 novel therapies. In the process of identifying targets for therapy, our understanding of the molecular pathways involved in malignancy has also increased. Several novel agents including bevacizumab, erlotinib, and cetuximab have proven that these agents can prolong the lives of patients with advanced NSCLC.

Understanding mechanisms of tumor cell growth and survival has translated into clinical trials of drugs that have changed the treatment landscape. The most recent NCCN guidelines now reflect these advances. First-line therapy for patients with metastatic disease or recurrent NSCLC and good performance status include four treatment options: chemotherapy alone, bevacizumab with chemotherapy, cisplatin with pemetrexed, or cetuximab with vinorelbine and cisplatin.

## Competing interests

The authors declare that they have no competing interests.

## Authors' contributions

All authors participated in drafting and editing the manuscript. All authors read and approved the final manuscript.
